# Interstitial Gingivitis

**Published:** 1908-10-15

**Authors:** Edward Cornelius Briggs


### INTERSTITIAL GINGIVITIS.

EDWARD CORNELIUS BRIGGS. M.D.

Read in the Section on Stomatology of the American Medical Association at the Fifty-Ninth Annual Session, held at Chicago, June, 1908. Printed in "The Journal of American Medical Association," Aug. 1, 1908.

While the term "Rigg's Disease" conveys a pretty definite meaning to the profession and to a majority of the laity, many of the profession have felt that it was not a term of sufficient scientific import and rather tended to emphasize the poverty of nomenclature in the dental department of medicine.

This led to the search for a more scientific name, and "pyorrhea alveolaris" became almost universal. This term, being descriptive of but one of many symptoms, failed to satisfy most of the students of this disease, and it remained for Talbot to point out that in all cases there was an underlying inflammation of the connective tissue of the gingiva and to suggest the name of " interstitial gingivitis."

### CAUSE.

The cause of interstitial gingivitis is still a question of dispute, although many theories have been put forward, some, if not all, of utmost plausibility. The truth of the matter probably is that the causes are many, or rather, that many are the conditions in the economy which give rise to the disturbance in the nervous and circulatory system producing the breaking down of the tissues about the roots of the teeth.

That it is a local expression of systemic disorder seems to me the best solution, and this is the opinion reached by many of our best observers. The opponents of this theory argue that as the disease does not exist where there are no teeth, it is, therefore, a purely local disease. This seems to me a specious argument, which if carried out to the fullest extent would admit the existence of few if any diseases of general or systemic origin when the result of that disease expresses itself in definite localities. Take, for instance, gangrene of a toe: Would the absence of that toe and, therefore, of disease in that member preclude the possibility of the organic malady which tends to gangrene.

By many observers a connection has apparently been traced between interstitial gingivitis and rheumatism; this is in turn refuted by those who find cases in which no rheumatism can be traced. It is my belief that both are but expressions of metabolic disorganization.

Various other constitutional diseases, such as renal, heart and liver disturbances, have been charged with the paternity of interstitial gingivitis. Each disease has in individual cases been apparently proved guilty, but the very failure at other times to find the result and the alleged cause associated seems to prove that back of them all is a common cause.

In the attempt to find such cause it may be well to see if it is possible to trace a condition precedent to these diseases and common to them all.

Goldthwaite, in his investigations of rheumatism, finds more than a coincidence in the presence of pus as an antecedent in many cases of rheumatism. Elimination of pus from the frontal sinus, antrum, tonsils and alveolar regions has given great relief in his cases.

Many grave renal diseases, while causing untold misery from the fact that the disorganized kidneys can not eliminate toxic and irritating products of metamorphosis, are, according to one authority, caused by irritation from products of faulty metabolism, due to imperfect or improper digestion in the alimentary canal.

Talbot, writing on autointoxication, says that interstitial gingivitis is found in full sway in Bright's disease, diabetes, rheumatism, gout, asthma, nervous disorders, etc., before these diseases have become of sufficient importance to be observed by the physician.

Fletcher, the advocate of thorough mastication, who holds the distinction of having created a new word in our vocabulary, namely, "to Fletcherize," maintains that if his precepts are followed and perfect digestion thereby produced, muscular pain or soreness can not occur. Muscles may become fatigued and refuse to act, but the sensations of cramp or lameness which most people feel after prolonged or unusual exertion exist only in those cases where there has been either improper digestion or faulty elimination or both.

The conclusion, therefore, seems to follow that the ultimate cause of interstitial gingivitis is the common cause of many chronic organic diseases, that is, toxins in the intestinal canal, whether they be poisons absorbed from some external source or thrown off as a result of physiologic chemical action on food good in nutritive quality but wrong in quantity or improperly prepared for the alimentary canal.

Cause and effect: What a problem it is to prove which is which! If interstitial gingivitis occurs about a malposed tooth, is it simply because the tooth is there and because its position harbors the disease? Or has the malposition led to improper mastication, faulty metabolism and the breaking down of interstitial tissues at a weak spot?

Does the deposit of calcareous substance on the teeth cause interstitial gingivitis? Or has a weak and flabby mastication produced autointoxication, permitted the deposits to form, and thus presented a nidus for the interstitial inflammation?

It is my belief that the first cause lies in the intestinal tract and that given perfect conditions there will be no interstitial gingivitis. Mark the words, "perfect conditions"—the various and often comparatively slight causes which contribute to imperfect conditions will be touched on in the section of the paper devoted to treatment.

Intestinal putrefaction produces indican in the urine and as indican will always be found in excess in cases of pus formation, it seems logical to charge the indican in the tissues with being largely responsible for the condition which leads to the breaking down into pus.

As causes of intestinal putrefaction Fossume cites the following:

1. Improper mastication, resulting in gastritis with lessened hydrochloric acid.

2. Gastritis, the lessened hydrochloric acid stimulating pancreatic secretions. In such cases delayed digestion carries the peptones too far down the tract to be absorbed and the coli communis changes it to indol, skatol, etc.

3. Excess of sugar, due to catarrh following fermentation and accompanied by much mucus so that digestion is delayed by (a) impoverished juices and (b) mechanical obstruction caused by presence of too much mucus.

4. Excess of proteids, with normal digestion.

5. Too much fat in food, mechanically interfering with absorption, though the digestion and the quantity of proteids are normal.

6. Slow digestion, with normal quantity of proteids.

7. Lack of bile, thus favoring putrefaction, as bile is strongly antiseptic.

8. Weak muscular action of the intestines.

Talbot lays great stress on autointoxication as a cause of this disease and has pretty conclusively shown that the uric acid habit is not a cause of interstitial gingivitis, but, like interstitial gingivitis, is only an expression of disturbed nutrition.

As has been said, observers have always shown a tendency to connect interstitial gingivitis with other systemic diseases—diseases whose origin is generally conceded to be due to faulty metabolism. Hence we find one man attributing the disease to rheumatism, another to gout, another to diabetes, etc., ignoring the fact that all these diseases have a first cause, which is faulty metabolism, and that, given the first cause, interstitial gingivitis becomes established, even in very young children (as scurvy) before the other diseases are diagnosed.

The treatment may be divided into, first, preventive and, second, curative, and each subdivided into local and systemic.

### PREVENTIVE TREATMENT.

*Local*—Inasmuch as it has been shown that the blood vessels of the gingiva become choked with the products of metabolism, it becomes an absolute necessity that the circulation should be assisted in every way possible.

Hence the advantage is seen of massage, of mildly stimulating mouth washes and, above all, of the stimulation resulting from the hand-polishing system for which Smith is the sponsor.

The patient should be instructed in the use of the tooth brush, it being a well-known fact among careful observers that many who brush their teeth faithfully many times a day never really clean them.

Inflammation of the mucous membrane, due to malocclusion or impaction, calls for the necessary correction, as such irritation easily becomes the starting point of interstitial gingivitis. This, perforce, leads to much regulating of the teeth, which was formerly neglected when there was no visible deformity, and to regulating at a much earlier age, since interstitial gingivitis, given the necessary condition for development, is no respector of age.

And this again leads to the subject of Fletcherizing, or the thorough and proper use of the teeth. Without their proper physiologic and anatomic use, all organs and members of the body tend to waste away or become a prey to disease, and the teeth are no exception. It, therefore, becomes evident that even a perfect set of teeth perfectly cared for can not be maintained in perfect health without perfect use.

No stomatologist fulfils his mission who, thorough though he may be in his technical care of the oral cavity, fails to hammer into the mind of his patient the paramount necessity of the thorough use of the teeth in mastication.

*Systemic*—I fancy that any one who has followed me thus far recognizes that I at least believe that the starting point of interstitial gingivitis lies in the alimentary canal and that faulty metabolism, whether due to imperfect digestion and assimilation of true foods or to the ingestion of substances not properly classed as foods, such as alcohol, makes for a condition of the interstitial tissue which lends itself easily to inflammation.

As Talbot has reminded us, the blood vessels in the alveolar process can not readily expand to the pressure of the blood, and if that blood be charged with toxins, then with high pressure and sluggish circulation the breaking down into inflammation becomes easy. Thus physicians are brought face to face with the importance—yes the necessity—of teaching temperance to their paticnts. Overeating and overdrinking are bound to fill the system with ptomains and ptomains largely of the toxic variety, and perhaps the worst thing about overdrinking is that it leads to overeating.

It should not be imagined that the small eater is necessarily not an overeater. He often eats some special kind of food to excess, as witness the sailors on long voyages who eat salt pork (and often not heartily) and become affected with scurvy (interstitial gingivitis).

Infants improperly or scantily fed have scurvy; and sometimes with plenty of variety at hand a man, through some perversion of taste, persists in eating some particular kind of food which the system can not properly eliminate . Tea, coffee, alcohol and tobacco all have their charm, and almost every adult is a devotee of one or more of these stimulants. They are not foods; in fact, each to a greater or less degree, according to its use or abuse, becomes a factor in upsetting the physiologic balance. The patient then, must be taught that if he dance he must pay the piper. At the age of thirty, when the anatomic structure is complete, there is not the demand for tissue building that there was in youth. All material not necessary for the repair of the material wear and tear of the machine consequently beccmes a clog in the system and interferes with the circulation. I have found that two meals a day are amply sufficient for a fairly strenuous life the last twenty years. Again I call attention to Fletcher and the tests of his endurance as made at Yale. Youths should eat proper food properly, and adults should refrain from overeating and eating in ruts. Though the diet may be simple, yet its simplicity should contain a variety. If there must be a cereal every morning, it should be varied from one day to another—not a difficult matter in these days of so-called "breakfast foods." And so with any article of food, constant and continued repetition should be avoided.

### CURATIVE TREATMENT.

*Systemic*.—For the patient presenting himself with a well-established case of interstitial gingivitis it is necessary to pursue an active and somewhat drastic course, both systematically and locally. The systemic treatment will be considered first.

To clean out the intestine I prefer to use podophyllin in centigram doses, giving two at night, followed by a saline on arising; then, to purify the intestinal canal, an intestinal antiseptic tablet made up of the sulphocarbolates of zinc, lime and soda, one at noon, another at night. The phodophyllin is repeated the second night and the rest of the treatment the next day, and so on until the physician is reasonably satisfied that the bowel has been thoroughly scoured and purified. This should be followed up with phosphate of soda, 60 centigrams, *t. i. d.* for an indefinite period, or until the local treatment is finished and the patient put on the general preventive treatment.

If there is much soreness of the gingiva or dark red spots, tender on pressure, indicative of a gouty tendency, salicylic acid or aspirin, 30 centigrams, *t. i. d.*, will often act magically. In obstinate cases it is very well to try iodid of potassium, 30 centigrams, *t. i. d.*, not only because the presence of iodin in the system acts most kindly in all cases of inflammation of mucous membrane, but also because the physician can never be entirely sure that a specific taint is not complicating the case. The usefulness of iodine in mucous inflammation, and also the fact that interstitial gingivitis may often be the first indication of arteriosclerosis, make hydriodic acid a timely remedy in such cases.

Calcium sulphid, an old remedy for sterilizing the system and checking the formation of pus, has of late received renewed attention from practitioners who have found it possible to get a pure preparation. It seems to be a clinical fact that saturation of the system with calcium sulphid, about gm. 20 to gm. 30 in twenty-four hours, will check the formation of pus in both alveolar abscess and in interstitial gingivitis. From my own observation and practice I am convinced of its efficacy.

The opsonic index has been taken in this disease. Treatment in accordance has been given a trial and favorable results reported.

The patient should be commanded to chew his food thoroughly, eating only what he can so chew.

The diet should be regulated, correcting the patient's tendency to take certain foods to excess and adding to his diet wholescme foods which he has eschewed owing to dislike or a fancied belief that they disagree with him. If the patient is over thirty the amount of flesh food ingested should be limited and the use of underdone meats discouraged. Almost invariably, on inquiry, it will be found that patients with interstitial gingivitis drink but little water. The deleterious effect of this habit in cases of interstitial gingivitis is too obvious to need further elucidation to the professional mind, but it is a condition too often overlooked. Six to eight glasses of water a day is little enough, yet few are they who drink as much. Obvious also is the importance of fresh air, exercise and deep breathing; yet these simple means for the establishment of good circulation are too often slighted or, because so obvious to the professional mind, their value is left for the patient to discover for himself.

To him the observance of these hygienic rules means simply an increase of his appetite, the indulgence of which in nine cases out of ten is just what should be avoided. It is elimination which should be sought, not further ingestion.

Old age, Metchnikoff says, is caused by the putrefaction of food in the colon. Measures to arrest or prevent this putrefaction tend to prolong life. He recommends lactic acid ferment and suggests buttermilk as a simple form of treatment.

Based on his suggestions, there are now on the market tablets of lactic acid ferment which, it is asserted, produce a clean, sweet colon and hence freedom from arteriosclerosis. I have been trying the effect of these with apparently good results. Patients will be found with well-marked interstitial gingivitis whose diet seems most correct. The patient may avow that he has a perfect stool with daily regularity, but on close questioning will admit that the stools are foul. It is in such cases that Fletcherism is demanded, for it should be remembered that Fletcher maintains that his method produces a stool absolutely free from odor. As a matter of fact these patients admit in nine cases out of ten that they bolt their food.

While realizing the importance of the practice of thorough mastication in these patients, it is well to remember the excellent temporary effects of intestinal antiseptics. Various writers have called attention to indications for treatment of the alimentary canal from observations of the condition of the tongue; for instance, a broad, thick, pallid tongue calls for alkaline treatment, both locally and systemically, and a pinched, dry, red tongue is evidence of need of acid treatment.

*Local.*—While the treatment up to this point has called for the keen attention and skill of the stomatologist, it is now that he has the final word and here that his skill and faith in his works bring about the real cure. For with interstitial gingivitis once established nothing will avail without the most thorough, persistent and intelligent local treatment.

Riggs who wrote on this disease and to whom all honor is due (although I do shrink at giving the unscientific name of Riggs' disease to interstitial gingivitis), laid the greatest stress on the necessity of thoroughly scaling the teeth. Younger, who has done more for the cause than any other living man, has hammered at this point most insistently. He says that the teeth must be scaled and that if to scale one tooth properly takes all of a long sitting the time must be so occupied and the one tooth done most thoroughly. He, as well as Riggs, has given the profession a set of scalers of great value to the operator. There are many others, notably Harlan, who have emphasized the importance of thorough scaling and devised instruments to facilitate the removal of deposits. I only wish to say that they have not put the matter forcefully enough. Those who have had experience in transplanting and replanting teeth know that a tooth should not only be clean and free from tartar, but that it must be smooth and polished. Many of the scalers of finest temper and cleverest design fail to leave the tooth in this condition and, therefore, they fail. The set of instruments invented by C. M. Carr do not scrape or draw-shave the teeth but plane them. When the tooth has been thoroughly planed by these instruments it is as smooth as glass. The varying lengths of operating shafts, curves, angles, etc., in devising which Carr has shown his genius, make it possible to reach every part of all teeth and their roots. He naturally emphasizes the importance of the perfect smoothness and cleanliness of the roots. In individual cases he can probably make his position good. His error is in considering the matter closed at that point and in not going deeper into cause and prevention. To sum up the instrumentation or operative treatment in interstitial gingivitis I do not hesitate to state that if with the teeth *in situ* the operator can produce the same freedom from deposits and the same polish that he could with the tooth in his hand and a cuttlefish disk in his engine, then he may be sure of curing for the time being any case of interstitial gingivitis. It is for the progressive operator to find the ways and means for doing this.

For the comfort of the patient and to enable the operator to work with deep and sure effect, it is almost a necessity to inject an anesthetic. Following are two good prescriptions for local anesthesia:

| R. | gm. or c.c. | | |
|---|---|---|---|
| Eucain hydrochloridi (B) ....... | 1 | 30 | gr. xx |
| Chloretone .................... | 1 | or | gr. xvss |
| Fluidextracti hamamelidis, ad....120 | | | fl℥iv |
| M. et sig.: For hypodermic use | | | |

| R. | gm. or c.c. | | |
|---|---|---|---|
| Cocainae hydrochloridi.......... | | 90 | gr.xiv |
| Chloretone .................... | 1 | or | gr. xvss |
| Fluidextracti hamamelidis, ad ...120 | | | fl℥iv |
| M. et sig.: For hypodermic use. | | | |

The eucain solution is for those who dread cocain or where the operator fears its effects, the cocain being perhaps surer in its anesthetic effect.

It is for the operator always to remember that if for any reason he can not perfect the smoothness of the tooth *in situ*, it remains for him to extract, polish and replant. While the concensus of experience shows that such teeth are apt to become subjects to absorption and come out after about seven years, yet I have cases of perfect teeth after a lapse of twelve years since replantation. It is impossible to place too much emphasis on the importance of the fixation of the teeth after such an operation, and also of teeth (not replanted) that have been loosened by the disease. Splints for this purpose are designed for temporary or permanent use, according to the necessity of the case.

It is now over ten years since I first made the assertion that, in proportion as teeth afflicted with interstitial gingivitis are in serious condition, in just that proportion is the removal of the pulp a necessary preliminary treatment.

Time and experience have only emphasized the conviction, and it is advised that the pulp be surgically removed where the disease has involved the root for at least half its length. In the majority of such cases the pulp will be found in an irritable condition, conducive to pulp stones or exostosis, and in the case of multiple roots one or more are often found with the pulp dead. The removal of the pulp by pressure anesthesia, as previously described by me,* is easy and simple.

Remedies and medicines in treatment must now be considered. The cleansed pocket needs something to stimulate the soft tissues to new granulation and to contract and heal around the root. Lactic acid in 50 per cent. solution is excellent and deserves trial. Aromatic sulphuric acid should always be in mind, especially if the disease has gone far and involved the alveolar process. They both promote the throwing off of any diseased bone tissues. I have found a mixture of carbolic acid and caustic potash, in equal parts, of greatest benefit in destroying soft tissue that is not granulating well or where there are exuberant granulations.

Iodin or compounds of iodin, as iodid of zinc or iodid of silver, have a most beneficial effect, the compounds especially adding to the well-known beneficial action of iodin the astringent and antiseptic effect of the metals. At this stage of the treatment also trichloracetic and monochloracetic acids are useful. If the disease has not gone deep enough to warrent the removal of the pulp and yet exposes dentine that is very sensitive, nitrate of silver for the posterior and chlorid of zinc for the anterior teeth have their well-known uses. Adrenalin and chlorid of aluminum are excellent astringents to tone and constrict the soft tissues.

For the patient to use at home, the free application of bicarbonate of soda is prescribed for its well-known effect both on inflamed mucous surfaces and its obtunding effect on sensitive dentine.

Limewater, fallen somewhat into disuse, is a splendid alkaline astringent and detergent remedy. Salt, free and in solution, has often proved efficacious in reducing inflammation.

---

*_Internat. Dent. Jour._, April, 1891.

Mouth lotions made up of several carminatives in alcoholic solution do much to stimulate and tone sluggish mucous surfaces. The incorporation of perborate of soda in a tooth powder does much to keep the parts clean and aseptic.

Interstitial gingivitis is a preventable and curable disease, and the stomatologist who does not prove this fact to his patient fails in his duty.

DISCUSSION.

DR. M. L. RHEIN, New York: I have for a long time been convinced that, while there is a well-marked condition known as gingivitis, when the alveolar tissues themselves are involved we should come nearer the truth if we would term that condition alveolitis rather than gingivitis. Both forms of the disease are present at times, but on the other hand gingivitis is sometimes present when alveolitis does not exist. The question of nomenclature ought to be settled. I wish that I could thoroughly agree with Dr. Briggs in attributing organic troubles entirely to faulty metabolism. I agree with him that faulty metabolism plays an important role in this condition. I have in this respect repeatedly recognized the value of what is known as Fletcherism. But, to illustrate the fact that faulty metabolism does not always play the initial role as the predisposing cause of this condition, we have renal trouble in children. This is not a question of faulty metabolism, the initial etiologic factor being the rapid development of the child physically. In such cases alveolitis often appears and urinalysis will show albumin, etc., Faulty metabolism may also be present, but only as a symptom, not as an etiologic factor. We should endeavor in our nomenclature to use great care in arriving at the true etiologic factor.

DR. EUGENE S. TALBOT, Chicago: I take issue with Dr. Rhein's views in regard to the etiology of this condition.

He contradicts himself; the very fact that the child has had renal trouble and associated conditions shows so far as we understand pathology to-day, that those conditions are due to faulty metabolism, to want of proper assimilation and adequate distribution of the food to the tissues. Fletcherism should be taught in dental colleges. Oral hygiene and Fletcherism should be so impressed on the minds of the students that when they commence practice these will be the watchwords constantly in mind. Some of the best medical schools are already teaching Fletcherism, and it is considered one of the most important studies in connection with digestion and assimilation. The treatment, both constitutional and local, suggested by Dr. Briggs, is the best that can be recommended with our present knowledge of materia medica.

DR. N. S. HOFF, Ann Arbor: There is no serious disease that is more easily curable than interstitial gingivitis. It is curable in any stage, unless the extraction of the teeth is clearly indicated. Like many other diseases, however, the condition may recur. The whole subject of the treatment of this disease and the practice of oral prophylaxis is going to elevate the standard of all other operations. More than any thing else that has come into dentistry in the last twenty years, the prophylactic treatment of Dr. Smith, that so many are using now, has elevated the standard of all operative procedures, and Fletcherism is going to help. Oral prophylaxis makes it imperative that we correct the malpositions of the teeth, and usually this is the first thing to do. We must retain all teeth possible to be retained, and keep each tooth in the best possible condition. This means practical oral prophylaxis and the cure of pyorrhea. This will finally revolutionize our system of crown and bridge work. The dentist of the future will have his hands full; he will have wonderful work to do. And if we are going to do what is expected of us in the future, how much is going to be expected of the dental college in the future! The course must be longer than three, four or five years for adequate dental preparation, and how we are going to get in a medical course with the dental is one of the things I can not understand. Our ideals are becoming so high and the demand so great that in the future we shall have to specialize in dentistry more than ever before. We shall in a few years have more specialties in dentistry than medicine has to-day; no one man can do all the work required to be done even now, and much less in the future.

Dr. Adelbert H. Peck, Chicago: Hypodermic injections in inflamed areas must be used with the utmost care, and the medicaments should never be carried into the area of pus accumulation. I have seen most dire results follow such mistakes. The fixation of loose teeth is one of the most important matters to engage our attention in this connection. When the teeth are loose they should be fixed by some mechanical means so that they will be firmly held in position, thus avoiding continual irritation to the diseased parts in their daily use. The list of drugs Dr. Briggs has given is splendid. Iodin, in proper combination, is one of the best agents to be used in these conditions.

Dr. N. S. Hoff, Ann Arbor: In regard to injecting cocain for operations on abscessed teeth, as suggested by Dr. Power, I am in the habit of injecting into the peridental membrane entirely and not into the gum tissue at all. This method is very satisfactory to me. If I cannot get the syringe point into the membrane I take a small Gates-Glidden drill in the right angle, make a small drill-hole alongside the root, and through this inject into the membrane only one or two drops. In that way I run little risk, and have never had any trouble.

Dr. Edward C. Briggs, Boston: As to "faulty metabolism" not being a blanket to cover all the conditions, it depends on what is meant by "faulty metabolism." The baby who does not get enough fat in the mother's milk (the rest of the food being right), is perhaps normal in its metabolism, but suffers because something is left out. Again, a well-nourished individual whose colon becomes subject to putrefactive changes may be all right in every other respect. He eats well, digests well, has normal dejecta daily; his heart, kidneys, lungs, etc., on examination are found to be apparently almost perfect; the only thing we can find is a foul stool. Correct that and local treatment begins to have effect. I had intended to lay stress on the importance of fixation of loosened teeth in interstitial gingivitis. It is absolutely necessary to devise some splint for temporary or permanent purposes.

